# Physiological amplitude: a systems-level framework for adaptive capacity in aging and metabolism

**DOI:** 10.3389/fragi.2026.1837722

**Published:** 2026-06-02

**Authors:** Grazyna Sepczynska

**Affiliations:** 1 Independent Researcher, Toronto, ON, Canada; 2 Eye Institute, Czestochowa, Poland

**Keywords:** aging, circadian regulation, metabolic resilience, mitochondrial function, network physiology, physiological amplitude, physiological variability, redox biology

## Abstract

**Background:**

Aging is characterized by progressive loss of physiological complexity, inter-system coordination, and adaptive capacity. While mitochondrial dysfunction, metabolic inflexibility, impaired stress responses, and circadian dysregulation are well established, an integrative systems-level construct linking these processes remains lacking.

**Objective:**

To introduce physiological amplitude as a unifying framework describing the bounded dynamic range of coordinated variability across interacting physiological systems.

**Methods:**

We develop a conceptual, hypothesis-generating framework grounded in network physiology and systems biology, integrating mitochondrial function, metabolic flexibility, hormetic stress responses, and circadian regulation into a unified architecture of system-level adaptation.

**Results:**

Physiological amplitude is defined as the bounded dynamic range within which coordinated multiscale physiological dynamics are maintained. Aging is conceptualized as a progressive contraction of this accessible range across interconnected biological networks. This framework distinguishes physiological amplitude from variability and resilience by positioning it as a higher-order property emerging from constraint-defined interactions across physiological systems.

**Conclusion:**

Physiological amplitude provides an integrative framework for interpreting multiscale physiological dynamics and generates testable hypotheses for quantification using continuous monitoring and network-based analysis. As a candidate systems-level descriptor, it may support the development of integrative biomarkers of aging and metabolic decline, pending empirical validation.

## Graphical Abstract

**Figure:**
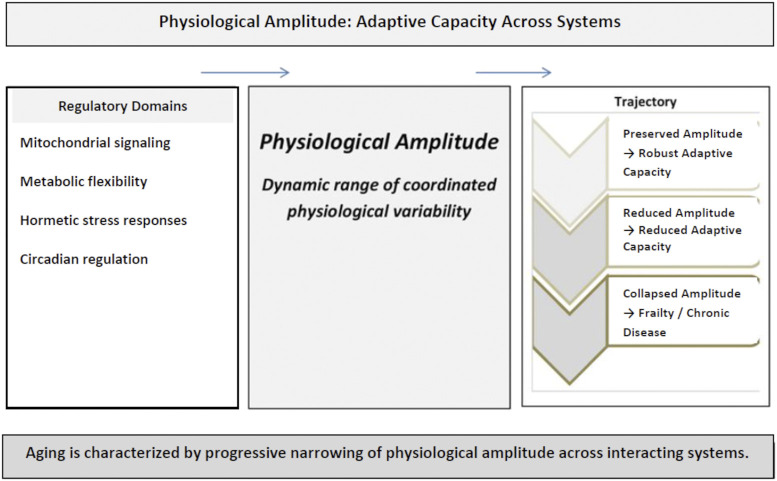
Physiological amplitude describes the dynamic range of coordinated variability across interacting physiological systems, integrating mitochondrial function, metabolic flexibility, stress adaptation, and circadian regulation into a unified model of adaptive capacity. Aging can be interpreted as a progressive contraction of the accessible physiological state space across interacting systems, thereby providing a basis for quantifying system-level decline using multiscale physiological data.

## Highlights

- Introduces physiological amplitude as a systems-level descriptor of adaptive capacity.
- Defines adaptive capacity as a bounded dynamic range of coordinated physiological dynamics.
- Integrates mitochondrial function, metabolic flexibility, circadian regulation, and hormesis.
- Reframes aging as progressive contraction of physiological dynamic range across interacting systems.
- Provides a pathway toward multiscale quantification of system-level physiology.

## Introduction

A systems medicine perspective recognizes that human physiology emerges from dynamic interactions among organ systems rather than isolated pathways. Network physiology demonstrates that biological function emerges from coordinated signaling across multiple spatial and temporal scales (Ivanov et al., 2016; Bartsch et al., 2015).

Within this framework, mitochondria function as integrative nodes linking bioenergetics, redox signaling, and stress adaptation (Nunnari and Suomalainen, 2012; Picard et al., 2016). Metabolic flexibility enables dynamic substrate utilization in response to energetic demands and environmental conditions (Goodpaster and Sparks, 2017; Muoio, 2014). Hormetic stress responses activate conserved pathways that enhance repair and adaptive capacity (Calabrese and Mattson, 2011; Rattan, 2008), while circadian systems provide temporal organization aligning metabolic processes with environmental cycles (Peek et al., 2013; Bass and Takahashi, 2010). Together, these domains form an integrated regulatory network supporting systemic metabolic resilience.

Aging can be conceptualized as a progressive loss of physiological complexity, inter-system coordination, and adaptive capacity, consistent with integrative geroscience frameworks and the hallmarks of aging describing interconnected biological drivers of functional decline (Kennedy et al., 2014; López-Otín et al., 2013). Within this context, physiological amplitude is introduced as a systems-level construct integrating these processes within a unified framework.

Conceptual frameworks play a critical role in systems medicine by defining integrative, testable constructs that guide empirical investigation across complex biological networks. Such frameworks are not intended to replace mechanistic models, but to provide structures for interpreting multiscale physiological data and generating testable hypotheses.

Physiological amplitude refers to the accessible range of coordinated physiological dynamics across interacting systems and is proposed as a higher-order descriptor of adaptive capacity, integrating mitochondrial function, metabolic flexibility, hormetic stress responses, and circadian regulation into a unified systems-level representation. These domains are foundational but not exhaustive; the construct is intentionally system-agnostic and extensible.

To clarify its intended role, physiological amplitude is introduced here as a latent systems-level construct rather than a directly measurable variable. It is not proposed as a single metric with a fixed computational definition, but as an organizing principle describing the accessible range of coordinated physiological states under system-level constraints. Accordingly, the formulation presented in this manuscript should be interpreted as a conceptual mapping between observable features and an underlying system property, rather than as a finalized or standardized quantitative measure. Further work will be required to define normalization strategies, weighting schemes, and implementation across specific physiological datasets.

## Novelty clarification and conceptual distinction

Although physiological variability, resilience, and network complexity have been extensively studied, physiological amplitude represents a distinct higher-order construct. Variability describes fluctuations within signals, resilience captures recovery following perturbation (Whitson et al., 2016), and network physiology characterizes coordinated interactions and coupling among physiological systems (Ivanov et al., 2016; Bartsch et al., 2015).

In contrast, physiological amplitude defines the bounded accessible range within which structured multivariate dynamics occur across interacting systems. It represents a constraint-defined property emerging from integrated system behavior rather than a feature derived from individual signals.

Accordingly, physiological amplitude functions as a higher-order boundary condition that defines the limits within which coordinated physiological dynamics can occur.

This framing shifts interpretation from local signal behavior to global system capacity, enabling assessment of the accessible range of coordinated dynamics across interacting physiological systems—corresponding to the accessible region of physiological state space. Unlike constructs that describe variability, recovery, or complexity in isolation, physiological amplitude delineates the system-level space within which these processes operate, providing a framework that can be empirically approximated using multiscale physiological data and generating testable predictions regarding the onset, progression, and potential reversibility of system-level dysfunction.

## Positioning relative to existing frameworks

### Structured comparison with existing constructs

To further clarify the conceptual contribution of physiological amplitude, Table 1 provides a structured comparison with related frameworks.

**TABLE 1:** Structured comparison of physiological amplitude with related conceptual frameworks.

Construct	Primary focus	Level of description	What it explains	Key limitation	Physiological amplitude adds
Physiological variability	Patterns and magnitude of signal fluctuations	Single-system/signal-level	Describes fluctuations of individual physiological signals	Does not capture cross-system coordination or system limits	Embeds variability within a bounded, system-level adaptive range
Resilience	Recovery following perturbation	Dynamic response	Describes recovery	Does not define the range within which recovery can occur	Defines the accessible range within which resilience operates
Allostatic load	Cumulative physiological burden of chronic stress	System-level (static)	Quantifies cumulative physiological strain	Does not describe real-time system dynamics or adaptability	Interprets accumulated burden as contraction of adaptive range
Network physiology	Interactions and coupling among physiological systems	Multisystem/network-level	Describes coordination among physiological systems	Does not define system-level boundaries or limits	Introduces bounded dynamic range as a constraint on network behavior
Energy allocation/constraint models	Allocation of energetic resources	Mechanistic/resource-based	Explains energetic trade-offs	Does not directly capture emergent system dynamics	Defines accessible coordinated states under energetic constraints

Physiological variability, resilience, allostatic load, and physiological complexity each describe important aspects of system behavior but do not define system-level limits of adaptive capacity.

Physiological amplitude differs by explicitly characterizing the system-level limits within which these processes occur. Variability and resilience operate within this range, while chronic stress and allostatic load may contribute to its reduction.

Thus, this framework captures system-level capacity as an expression of integrated regulatory and energetic constraints acting across scales.

This framing enables interpretation of early system-level dysfunction as a loss of accessible adaptive range, even when individual physiological signals remain within normal ranges—an aspect not captured by existing variability- or resilience-based metrics.

While constraint- and allocation-based models describe how limited energetic resources are distributed across competing physiological processes, physiological amplitude characterizes the resulting accessible range of coordinated system behavior under those constraints. Importantly, systems with similar energetic capacity or allocation profiles may exhibit different physiological amplitudes depending on the degree of cross-system coordination and regulatory integration. In this sense, physiological amplitude captures the functional expression of constraint structure at the systems level, providing a complementary perspective that links resource limitation to observable patterns of coordinated physiological dynamics.

### Physiological amplitude

Physiological amplitude is defined as the bounded dynamic range of coordinated variability across interacting physiological systems within which stability and adaptive responsiveness are maintained.

In systems terms, this bounded dynamic range can be interpreted as the accessible region of physiological state space defined by underlying constraints.

Within this framework, health is characterized by structured variability that enables effective responses to internal and external perturbations. At the systems level, the breadth of this structured multivariate dynamics is captured by physiological amplitude.

This property emerges from the integrated function of mitochondrial activity, metabolic flexibility, stress adaptation, and circadian regulation. Disruption across these domains leads to reduced coordination, impaired responsiveness, and progressive narrowing of physiological amplitude, often preceding overt pathology.

Physiological amplitude is conceptually distinct from both variability and resilience. Variability describes fluctuations within individual signals, and resilience reflects recovery following perturbation.

This formulation defines the system-level range within which structured multivariate dynamics and recovery processes can occur. In practice, this construct can be approximated using measures of oscillatory dynamics, responsiveness to perturbations, and cross-system coupling across temporal scales. Advances in continuous physiological monitoring and network-based analysis provide emerging tools for such quantification.

**FIGURE 1:**
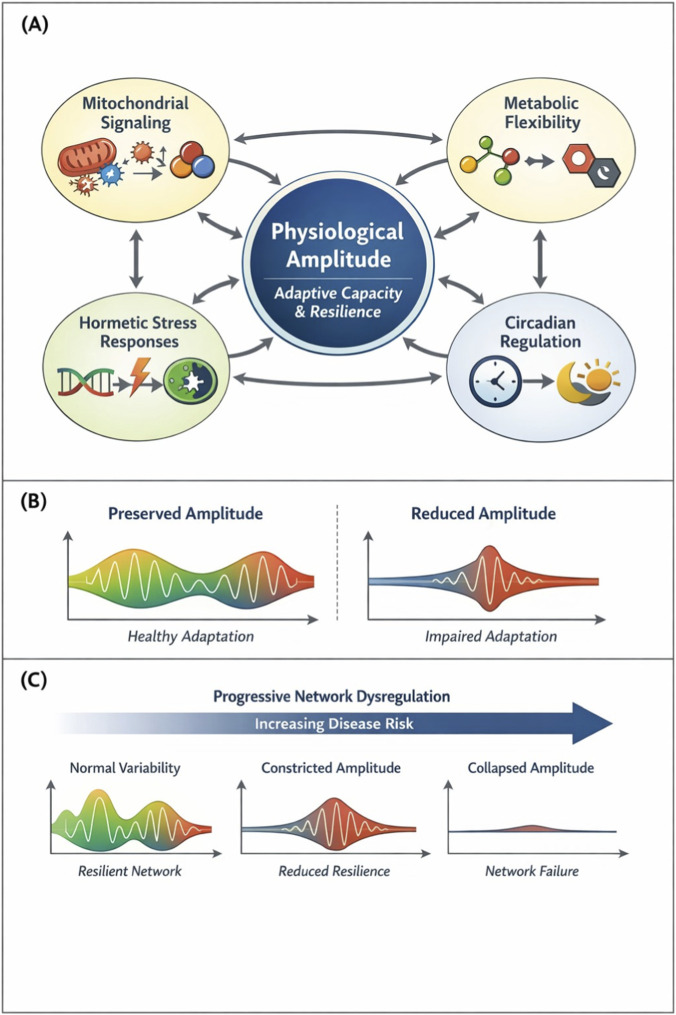
Systems-level framework of physiological amplitude and metabolic resilience. **(A)** Integrated regulatory network comprising mitochondrial signaling, metabolic flexibility, hormetic stress responses, and circadian regulation. Physiological amplitude emerges as a systems-level property from coordinated interactions across these domains and enables adaptive capacity and resilience. **(B)** Physiological amplitude represented as the dynamic range of coordinated variability over time. Preserved physiological amplitude is characterized by broad, structured oscillations reflecting robust adaptive capacity, whereas reduced physiological amplitude is associated with constrained variability and impaired adaptation. **(C)** Conceptual trajectory of progressive constraint of physiological amplitude associated with network dysregulation and declining systemic resilience. This progression illustrates a transition from preserved, reduced, and ultimately collapsed physiological amplitude, corresponding to increasing disease risk and reduced adaptive capacity.

As illustrated in Figure 1, physiological amplitude can be represented both as an emergent network property and as the dynamic range of coordinated physiological variability over time.

**FIGURE 2:**
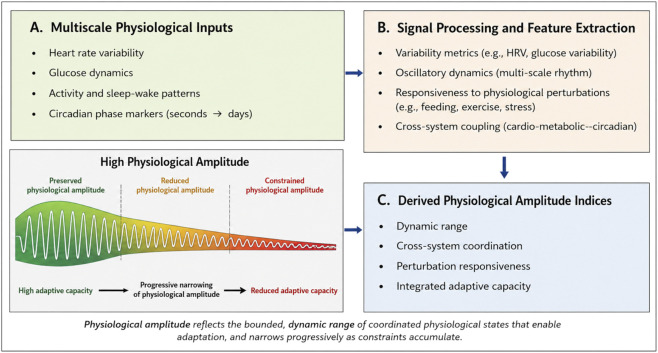
Operational framework for quantifying physiological amplitude. Figure 2 illustrates the operationalization of physiological amplitude as a systems-level construct through integration of multiscale physiological data streams and network-based analytical methods. Rather than proposing a new mechanistic model, this framework provides a structured mapping between the conceptual definition of physiological amplitude and measurable physiological signals across temporal and spatial scales. Figure 2 should therefore be interpreted as a methodological extension of the conceptual framework rather than a modification of its underlying theoretical structure. **(A)** Multiscale physiological data, including cardiovascular dynamics (e.g., heart rate variability), metabolic regulation (e.g., glucose dynamics), behavioral patterns (e.g., activity and sleep cycles), and circadian rhythms, provide continuous or repeated observations of system-level function. **(B)** Analytical processing captures features of physiological organization, including intra-system variability, oscillatory structure across time scales, responsiveness to perturbations (e.g., feeding, exercise, stress exposure), and cross-system coupling reflecting network coordination. **(C)** Integration of these features yields derived indices reflecting the bounded dynamic range of coordinated physiological behavior, referred to as physiological amplitude. These indices are intended to support hypothesis generation and future empirical validation in the context of aging and metabolic decline. Importantly, this figure represents an operational translation of the conceptual framework rather than a fully validated computational or diagnostic model.

## Discussion

This framework integrates mitochondrial signaling, metabolic flexibility, hormetic stress responses, and circadian regulation into a unified systems-level model of adaptive capacity. It is not intended to replace existing constructs, but to provide a unifying framework that defines the bounded dynamic range within which they operate. In doing so, it bridges signal-level measurements with emergent system-level behavior, linking observable physiological dynamics to underlying system capacity.

The framework aligns with Kitano’s concept of biological robustness (Kitano, 2004) and with bioenergetic constraint models emphasizing energetic limitations on physiological organization (Rolfe and Brown, 1997). Energy availability constrains the system, whereas physiological amplitude reflects the functional expression of those constraints.

This distinction can be further clarified in relation to constraint- and allocation-based frameworks. Energy allocation models describe how limited resources are distributed across competing physiological processes, whereas physiological amplitude characterizes the accessible range of coordinated system states that emerges under these constraints. In this context, energetic capacity, resource prioritization, and regulatory trade-offs define system boundaries, while physiological amplitude reflects their integrated expression at the level of whole-system behavior. Accordingly, it provides a higher-order description of system capacity emerging from constraint structure that complements, rather than replaces, allocation-based models (Rolfe and Brown, 1997; Schuler et al., 2026).

A conceptual example illustrates this distinction. During acute stress, physiological systems transiently shift toward a constrained region of the accessible range, followed by recovery to baseline, reflecting preserved amplitude. In contrast, chronic stress leads to sustained prioritization of specific physiological processes, reducing variability, weakening cross-system coordination, and progressively narrowing the accessible range. This transition reflects contraction of physiological amplitude rather than an isolated change in variability or resilience.

From a mechanistic perspective, constraints propagate across physiological systems through interconnected regulatory pathways. For example, reduced mitochondrial energetic capacity may limit ATP availability, constraining metabolic flexibility and impairing coordinated system responses to perturbation (Nunnari and Suomalainen, 2012; Wallace, 2012; Sun et al., 2016; Rolfe and Brown, 1997). This reduction in energetic throughput can weaken cross-system coupling and diminish adaptive responsiveness, leading to contraction of the accessible physiological state space and reduced system-level adaptability. In this way, local constraints at the level of cellular energetics scale to system-level reductions in physiological amplitude, linking mechanistic dysfunction to emergent loss of adaptive capacity.

At a minimal systems level, physiological amplitude can be interpreted through a hierarchical constraint structure. Mitochondrial energetic capacity defines the upper bound of accessible physiological states by determining the overall availability of bioenergetic resources. Circadian regulation provides temporal organization, structuring when physiological processes occur and enabling coordinated system transitions across daily cycles. Within these constraints, metabolic flexibility and hormetic stress responses determine how efficiently and adaptively the system can move within the accessible state space. Metabolic flexibility governs substrate utilization and energetic responsiveness, while hormetic mechanisms modulate adaptive capacity through stress-induced repair and reinforcement processes. Together, these domains define both the boundaries and the internal dynamics of physiological amplitude, linking cellular energetics to system-level behavior.

Within this framework, physiological amplitude should not be interpreted as an independent governing variable separate from underlying physiological processes, nor as a simple derived metric computed from observed signals. Rather, it represents an emergent systems-level property that reflects the integrated effect of underlying constraints. In this sense, physiological amplitude is best understood as a state-space descriptor of system capacity that arises from constraint structure, rather than a primary driver of system behavior. This distinction clarifies that the construct is descriptive but not merely phenomenological; it provides a structured representation of how mechanistic constraints manifest at the level of coordinated system dynamics.

This work is conceptual and intended to provide a structured framework for hypothesis generation rather than a fully validated quantitative model. The proposed operationalization represents an initial approximation, and further work is required to define standardized metrics, validate reproducibility across populations, and establish clinically meaningful thresholds. In addition, the simplified representations presented here do not capture the full complexity of nonlinear and time-dependent interactions that characterize physiological systems.

## Network physiology

Network physiology conceptualizes the organism as an integrated system of interacting physiological networks (Ivanov et al., 2016; Bartsch et al., 2015). Consistent with prior work on physiological complexity and fractal dynamics developed by Lipsitz, Goldberger, and colleagues (Lipsitz, 2002; Goldberger et al., 2002a; Goldberger et al., 2002b), healthy systems exhibit structured variability and dynamic coupling, while aging is associated with reduced variability and weakened coordination. At the systems level, physiological amplitude can be interpreted as an expression of network adaptability. Broad amplitude reflects coordinated integration, whereas narrowing reflects progressive decoupling.

## Core mechanistic domains

### Mitochondrial signaling

Mitochondria serve as central bioenergetic and signaling hubs linking metabolism, redox balance, and stress responses (Nunnari and Suomalainen, 2012; Wallace, 2012; Picard et al., 2016).

### Metabolic flexibility

Metabolic flexibility enables adaptive substrate utilization in response to changing energetic demands (Goodpaster and Sparks, 2017; Muoio, 2014).

### Hormetic stress responses

Hormesis enhances resilience through adaptive responses to transient stressors (Calabrese and Mattson, 2011; Rattan, 2008).

### Circadian regulation

Circadian rhythms coordinate physiological processes across daily cycles (Peek et al., 2013; Bass and Takahashi, 2010).

These domains are interconnected and collectively shape system-level adaptive capacity.

Within this framework, interactions among domains are best understood as context-dependent and partially hierarchical rather than strictly symmetric. While bidirectional coupling is a defining feature of network physiology, certain domains may exert dominant constraint effects under specific conditions. For example, mitochondrial energetic capacity may impose system-wide limits, whereas circadian regulation provides temporal structure for coordinated activity. The relative influence of these domains shifts dynamically with physiological state and environmental conditions. Physiological amplitude therefore emerges from the integrated effect of interacting constraints, reflecting not only variability and coupling, but the organization of system-level regulatory priorities and limitations.

## Clinical and translational implications

Physiological amplitude provides a framework for interpreting chronic disease as a progressive loss of adaptive capacity across interconnected physiological systems.

Early dysfunction may manifest as reduced variability and weakened coupling prior to structural pathology (Lipsitz, 2002; Goldberger et al., 2002a; Goldberger et al., 2002b). Disease progression can be conceptualized as progressive constraint of physiological amplitude over time.

Wearable technologies and continuous monitoring enable quantification of multivariate physiological dynamics in real-world settings, supporting translation into clinical tools.

In practice, this construct may be approximated using composite indices integrating variability, coupling, and responsiveness across systems, enabling early detection, risk stratification, and monitoring.

## Conclusion and future directions

Physiological amplitude is introduced as a systems-level descriptor emerging from coordinated interactions among key regulatory domains. By framing adaptive capacity in terms of bounded dynamic range, this framework provides a conceptual bridge between mechanistic biology and system-level function, expressed as dynamics within a bounded physiological state space. Future work should focus on operationalization using multiscale physiological data and validation across populations. As a candidate integrative descriptor, physiological amplitude offers a foundation for linking biological aging to functional decline across scales.

## Toward quantification of physiological amplitude

In practice, this construct may be approximated through multivariate indices integrating.

- Intra-system variability
- Multiscale oscillatory dynamics
- Perturbation responsiveness
- Cross-system coupling

These indices represent empirical projections of an underlying systems-level property and require validation and standardization.

A critical distinction in this context is between adaptive physiological variability and pathological instability. Increased variability within individual signals does not necessarily indicate preserved physiological amplitude. Adaptive states are characterized by coordinated, structured variability across interacting systems—manifested as preserved coupling, context-appropriate responsiveness, and organized dynamics across temporal scales—and are consistent with principles of biological robustness, in which functional stability emerges from coordinated system-level regulation rather than isolated signal behavior (Kitano, 2004).

In contrast, pathological states may exhibit elevated or erratic variability that is poorly coordinated, weakly coupled, and temporally disorganized. Such patterns reflect a breakdown of integrated regulation rather than expansion of adaptive capacity.

Accordingly, operationalization of physiological amplitude requires multivariate approaches capable of distinguishing coordinated system-level dynamics from unstructured or decoupled fluctuations.

Physiological amplitude may be approximated through composite representations of observable system features, for example, in the form *A ≈ f(R, C, S),* where A denotes the latent system-level property and R, C, and S represent measurable but partial projections of the latent construct.

- R = normalized dynamic range across physiological signals
- C = cross-system coupling strength
- S = adaptive responsiveness to perturbation

This formulation is intentionally non-definitive and should not be interpreted as a finalized computational model. Rather, it illustrates how observable physiological features may be mapped onto an underlying latent construct, providing a starting point for future operationalization.

Importantly, physiological amplitude is not equivalent to any individual component (R, C, or S), but reflects their integrated constraint-defined interaction at the systems level.

Accordingly, increased variability alone is neither necessary nor sufficient for preserved physiological amplitude; only variability that is coordinated, coupled, and responsive within system-level constraints reflects adaptive capacity.

To illustrate a potential implementation, this construct can be approximated using integrated physiological data streams. For example, normalized heart rate variability could serve as a proxy for dynamic range (R), while measures of temporal correlation or coherence between cardiovascular and metabolic signals (e.g., heart rate and glucose dynamics) could approximate cross-system coupling (C). Responsiveness to perturbation (S) could be estimated by quantifying system responses to defined inputs such as meals, physical activity, or stress exposure. A simplified composite index could then be constructed by integrating these normalized features into a multivariate function, providing an empirical approximation of physiological amplitude. While this representation is intentionally simplified, it illustrates how the construct may be translated into measurable quantities for hypothesis testing and future validation.

At its current stage, physiological amplitude should be interpreted as a conceptual, inferential construct whose empirical approximation depends on context-specific modeling choices rather than a standardized or directly measurable quantity.

## Illustrative comparison across physiological states

To clarify how the components of physiological amplitude interact in practice, this framework can be illustrated across representative physiological states.

In a healthy state, physiological amplitude is preserved and characterized by a broad dynamic range (R), strong cross-system coupling (C), and robust responsiveness to perturbation (S). For example, heart rate variability exhibits a wide and structured range, glucose dynamics remain tightly regulated with coordinated postprandial responses, circadian rhythms are stable and synchronized, and physiological systems respond efficiently to challenges such as meals, physical activity, or stress (Hawley et al., 2014). Together, these features reflect a coordinated and dynamically accessible range.

In early dysfunction, physiological amplitude is reduced. Dynamic range may be moderately diminished, cross-system coupling weakened, and responsiveness to perturbation blunted. For instance, heart rate variability may be reduced or less structured, glucose excursions may become more variable and less tightly regulated, circadian rhythms may show early signs of misalignment, and responses to perturbations may be delayed or less efficient. These changes reflect partial contraction of the accessible physiological range, even in the absence of overt disease.

In advanced dysfunction, such as metabolic syndrome or frailty, physiological amplitude is further constrained. Dynamic range is reduced, cross-system coupling is weak or disrupted, and responsiveness to perturbation is markedly impaired. This may manifest as low heart rate variability, erratic or poorly controlled glucose dynamics, disrupted sleep–wake cycles, and diminished physiological responsiveness to environmental or internal challenges. In this state, the accessible adaptive repertoire is substantially narrowed.

Importantly, these components are not independent or necessarily equally weighted. Increased variability within a single signal does not imply preserved physiological amplitude if cross-system coupling is weak or absent. Conversely, preserved coupling cannot fully compensate for severely reduced dynamic range or impaired responsiveness. Physiological amplitude therefore reflects the integrated and constraint-defined interaction among dynamic range, coordination, and adaptability, rather than any single component in isolation.

## Testable predictions

The framework generates the following testable predictions.

1. Greater metabolic resilience is associated with broader physiological amplitude.
2. Early dysfunction manifests as reduced variability and coupling.
3. Interventions increase physiological amplitude over time.
4. Mitochondrial function correlates positively with amplitude.
5. Consistent with frailty phenotype and deficit-accumulation models (Fried et al., 2001; Mitnitski et al., 2001), declining physiological amplitude may predict adverse outcomes.

## Data Availability

The original contributions presented in the study are included in the article/supplementary material, further inquiries can be directed to the corresponding author.
